# Draft Genome Sequences of Three Francisella tularensis subsp. *mediasiatica* Strains Isolated in the Altai Territory, Russian Federation

**DOI:** 10.1128/MRA.01202-19

**Published:** 2020-02-13

**Authors:** Alexander N. Mokrievich, Angelina A. Kislichkina, Tamara Y. Kudryavtseva, Raisa I. Mironova, Galina M. Vakhrameeva, Nina A. Shishkova, Vitalii S. Timofeev, Alexandr G. Bogun, Vitaliy M. Pavlov, Ivan A. Dyatlov

**Affiliations:** a State Research Center for Applied Microbiology and Biotechnology, Obolensk, Russia; Broad Institute

## Abstract

We report the draft genome sequences of three Francisella tularensis subsp. *mediasiatica* strains isolated in the Altai Territory, Russian Federation.

## ANNOUNCEMENT

Francisella tularensis is the etiologic agent of tularemia, one of the most pathogenic bacterial infections to humans. Previously, it was considered that F. tularensis subsp. *mediasiatica* exists only in a few regions of Central Asia (1). However, in 2011 in the Altai Territory of the Russian Federation, a natural source of tularemia was found in which circulating strains of F. tularensis subsp. *mediasiatica* were present (2). The following three F. tularensis strains isolated in the Altai Territory were studied in the reference center for monitoring tularemia: strain A-554, from the tick Haemaphysalis concinna, strain A-678, from the tick Ixodes persulcatus, and strain A-823, from spleen of the red vole (Myodes rutilus). All these strains were isolated using direct plating of homogenized samples on selective medium (FT agar [SRCAMB, Obolensk, Russia]) on petri dishes.

The species F. tularensis was confirmed using PCR with primers for the *fopA* and *iglC* genes (2). All three strains were virulent for BALB/c mice (certain lethal dose, <10 CFU) and sensitive to erythromycin (2, 3). We found that they were able to ferment glycerol and showed citrulline ureidase activity but not beta-lactamase activity (3). Therefore, we concluded that these strains belonged to F. tularensis subsp. *mediasiatica*. This was confirmed using the Chi1f primer according to the method described in reference 4. Multilocus variable-number tandem-repeat analysis (MLVA) for 25 (5, 6) and 15 loci (3) showed that each of the investigated strains (A-554, A-678, and A-823) had its own specific MLVA genotype, differing in the Ft-M3, Ft-M6, Ft-M7, Ft-M11, and Ft-M20 loci (3).

Here, we report three draft genome sequences of Francisella tularensis subsp. *mediasiatica* strains isolated in the Altai Territory.

The stocks of the strains were stored at –70°C in cryoprotective medium. Bacterial cultures were cultivated on FT agar (SRCAMB, Russian Federation) for 18 h at 37°C. DNA was isolated with a GenElute bacterial genomic DNA kit (Sigma-Aldrich, USA). Whole-genome sequencing was performed using an Illumina MiSeq instrument according to the manufacturer’s instructions. DNA libraries were prepared using a Nextera DNA library preparation kit. A MiSeq reagent kit v3 (300 cycles) was used for sequencing. For each genome, the reads without filtering were assembled *de novo* using SPAdes v3.11.1 (http://cab.spbu.ru/software/spades/). Finally, we obtained 101 to 106 contigs for each genome (Table 1). The genome sizes ranged from 1.82 to 1.85 Mb. The final assemblies were annotated with the NCBI Prokaryotic Genome Annotation Pipeline. Each genome contains 1,921 to 1,971 genes. The GC content of all samples was 32.3%.

**TABLE 1:** Strain-identifying information and basic statistics on assemblies and annotations

Strain name	Host	Raw data accession no. (SRA)	Assembly accession no. (GenBank)	No. of reads	*N*_50_ (bp)	Size (bp)	No. of contigs	Total no. of genes	No. of coding genes
SCPM-O-B-7175 (A-554)	*Haemaphysalis concinna*	SRR8573488	SGWP00000000	1,573,580	35,350	1,817,080	101	1,921	1,565
SCPM-O-B-7176 (A-678)	*Ixodes persulcatus*	SRR8573489	SGWO00000000	807,826	36,817	1,845,983	103	1,950	1,598
SCPM-O-B-7177 (A-823)	*Myodes rutilus*	SRR8573486	SGWN00000000	1,217,624	35,307	1,853,999	106	1,971	1,607

### Data availability.

The genome sequences and sequence reads were deposited in the GenBank/ENA/DDBJ databases under the accession numbers listed in Table 1.
